# A Novel Markov Model Projecting Costs and Outcomes of Providing Antiretroviral Therapy to Public Patients in Private Practices versus Public Clinics in South Africa

**DOI:** 10.1371/journal.pone.0053570

**Published:** 2013-02-06

**Authors:** Rory Leisegang, Gary Maartens, Michael Hislop, John Sargent, Ernest Darkoh, Susan Cleary

**Affiliations:** 1 Division of Clinical Pharmacology, University of Cape Town, Cape Town, South Africa; 2 Aid for AIDS, Medscheme Pty Limited, Cape Town, South Africa; 3 BroadReach Healthcare, Cape Town, South Africa; 4 Health Economics Unit, University of Cape Town, Cape Town, South Africa; University of Washington, United States of America

## Abstract

**Introduction:**

Providing private antiretroviral therapy (ART) care for public sector patients could increase access to ART in low- and middle-income countries. We compared the costs and outcomes of a private-care and a public-care ART program in South Africa.

**Methods:**

A novel Markov model was developed from the public-care program. Patients were first tunneled for 6 months in their baseline CD4 category before being distributed into a dynamic CD4 and viral load model. Patients were allowed to return to ART care from loss to follow up (LTFU). We then populated this modeling framework with estimates derived from the private-care program to externally validate the model.

**Results:**

Baseline characteristics were similar in the two programs. Clinic visit utilization was higher and death rates were lower in the first few years on ART in the public-care program. After 10 years on ART we estimated the following outcomes in the public-care and private-care programs respectively: viral load <1000 copies/ml 89% and 84%, CD4 >500 cells/μl 33% and 37%, LTFU 14% and 14%, and death 27% and 32%. Lifetime undiscounted survival estimates were 14.1 (95%CI 13.2–14.9) and (95%CI 12.7–14.5) years with costs of 18,734 (95%CI 12,588–14,022) and 13,062 (95%CI 12,077–14,047) USD in the private-care and public-care programs respectively. When clinic visit utilization in the public-care program was reduced by two thirds after the initial 6 months on ART, which is similar to their current practice, the costs were comparable between the programs.

**Conclusions:**

Using a novel Markov model, we determined that the private-care program had similar outcomes but lower costs than the public-care program, largely due to lower visit frequencies. These findings have important implications for increasing and sustaining coverage of patients in need of ART care in resource-limited settings.

## Introduction

Expanding capacity to deal with the HIV epidemic is a formidable task in low- and middle-income countries given the scale of the epidemic and the limited public health infrastructure. While much has been achieved to make antiretroviral therapy (ART) affordable, access to care is still inadequate. According to the latest UNAIDS report, only 46% of those who were in need had started ART by the end of 2010 in low- to middle-income countries [1].

One way to expand access to ART and improve retention within ART care for public sector patients is to utilize the private sector. In many low- and middle-income countries a high proportion of doctors work in the private sector [2]. Contracting private doctors to initiate ART and follow up public sector patients in their private rooms according to the public sector guidelines has been successfully implemented in Botswana [2] and other developing country settings [3]. However, there are concerns about the ability and willingness of individual private doctors to implement the public health approach to ART management, and about high costs in the for-profit private sector. To date there have been no published comparisons of clinical and economic outcomes of the provision of ART care to public patients between the private sector and public sector.

In addition to the debates about public versus private ART care, there are also questions about how frequently patients should be followed up, and by whom. In the earlier years of ART provision, patients were required to attend facilities for regular consultations with doctors or nurses [4]. More recently, however, there has been a move towards less frequent follow-up, and towards task shifting from doctors to nurses, and from nurses to counselors [5]. It is however unclear whether this changing intensity in follow-up will impact negatively on patient adherence and outcomes.

We assessed the costs and outcomes of providing ART care for public patients in the private versus public sector in two South African ART programs where no co-payment from patients was required: a grant-funded program providing care for public patients in private practices and a public-sector program providing care for public patients in public sector community clinics. We utilized a newly developed Markov-model, which addresses many of the limitations of existing models [6].

## Methods

### Study design

We assessed the costs and outcomes of ART provision in the private-care and public-care models to provide care to public sector dependent patients. We took the provider's perspective and only included ART-related costs: antiretroviral drugs, CD4+ cell count (CD4) and viral load (VL) monitoring, toxicity laboratory monitoring, and public clinic or private general practitioner (GP) visits. We used Markov modeling to extrapolate primary data in order to estimate results over 10 years and lifetime for costs, rates of loss to follow-up and life years. Zero and three percent annual discount rates were used. The model was developed using data from the public-care cohort, and validated externally using data from the private-care cohort. Uncertainty was assessed using multi-way and probabilistic sensitivity analyses.

### Study setting

ART care for patients in both programs followed the 2003 South African national guidelines, which were based on the 2003 World Health Organization guidelines for resource-limited settings [7]. Patients were eligible for ART when they met the following criteria: either a CD4 below 200 cells/µL or a WHO stage 4 illness (other than extra-pulmonary tuberculosis) irrespective of the CD4 count. The first line ART regimen consisted of two nucleoside reverse transcriptase inhibitors (NRTI), zidovudine (ZDV) or stavudine (D4T) with lamivudine (3TC), with a non-nucleoside reverse transcriptase inhibitor (NNRTI), nevirapine (NVP) or efavirenz (EFV). Viral load and CD4 counts were monitored 6 monthly. Patients with confirmed virologic failure (two consecutive viral loads > = 5000 copies/ml) in spite of enhanced adherence promotion, were switched to a second line regimen of two NRTIs, ZDV and didanosine (DDI), in combination with a boosted protease inhibitor, lopinavir/ritonavir (LPV/r). Safety monitoring was limited to serum alanine aminotransferase (ALT), complete blood count, and lipogram for patients on NVP, ZDV, and LPV/r respectively.

### Cohort Description

The public-care cohort was the Khayelitsha HIV treatment program, which is a public sector program operating in an urban area in Cape Town, South Africa. The program is jointly funded by the state and a donor, Medecins Sans Frontieres. ART care was provided at three primary care clinics. ART was initiated by doctors but routine follow up was largely done by nurses. The clinics operated on a queue system and therefore patients would spend between 1–4 hours at the clinic. Counselors and peer-educators played an important role in educating and encouraging patients while they waited to see clinical staff. Most patients returned to the clinic every month to collect medicines, attend group or individual counseling sessions, and/or for clinical assessments. We included data from the inception of the program on 15 January 2000 until 25 Jan 2008.

The private-care cohort was the BroadReach Healthcare program, a donor-funded (President's Emergency Plan for AIDS Relief (PEPFAR)) managed-care ART program. Patients were recruited into the program at several urban and rural public sector clinics in the Mpumulanga, Eastern Cape and Kwazulu-Natal provinces in South Africa. ART care was provided by local contracted general practitioners (GPs) in their private practices on an appointment basis and visit frequency was pre-specified. The private doctors had to successfully complete internet-based training on the national ART guidelines before they could enroll patients. Telephonic counseling support for the patients and clinical guidance for the doctors was provided by Aid for AIDS, a private sector disease management program. Patients collected their medication from the doctors' rooms monthly, but clinical consultations were performed less frequently. We included data from the inception of the program on 1 May 2005 until 31 July 2010. New patient enrollment was stopped in March 2008.

In both cohorts severely ill or complicated patients were referred to secondary level public sector hospitals for further management and then re-integrated back into the program once their condition had stabilized. Data were entered prospectively into databases. Deaths were ascertained by several mechanisms: (1) clinic staff or private practice practitioners who learnt of a death from family members or friends, would either complete a specific form and fax it to a central office or capture it on a computer-based system onsite; (2) staff and program administrators identified patients who had missed several appointments and contacted a family member or treatment supporter of the patient to determine whether the patient was deceased and if so the date of death; and (3) the patient's South African identity number, where available, was used to cross-reference the South African national death register to establish whether a death was recorded.

We included adult patients (19 years and older) who started first line ART within the programs and had a baseline CD4 count below 200 cells/µL. The study intervals differed somewhat for each cohort, although the median year of starting ART was 2005 in both cohorts. A patient's follow-up period was truncated on the date they either: transferred out of the program, died, on the study end date, or on the last date seen if they were not seen within six months of the end of the study period and their identity number was not available (and we were therefore unable to ascertain whether they had died).

### Healthcare utilisation and cost data

GP or clinic utilisation was determined from the electronic database records for both cohorts. The cost in South Africa Rands (ZAR) for a public-sector clinic visit was determined from a previously published estimate [4]. In that study, the unit clinic visit costs included time allocations for nurses, doctors, and counselors, and this has changed in more recent times due to increased task shifting. Together with improved economies of scale and learning by doing, cost would have fallen substantially had it not been for substantial increases in doctor's salaries over the same period. We therefore decided to only use the consumer price index table [8] to inflate costs to April 2010 levels. Private GP visit costs were determined from contracted rates in April 2010.

Drug utilisation was divided into first line (2NRTIs and NNRTI) and second line (2NRTIs and PI) therapy, and the average utilisation of each drug was determined within each line of therapy. Because estimates of ARV drug utilization were not available within our dataset, we conservatively assumed that all patients had received their ARVs each month and therefore allocated full monthly ARV drug costs within the ART model. ARV drug costs were set at the public sector tender prices for April 2010.

There was some under-reporting of CD4 and VL monitoring, and ARV laboratory toxicity monitoring was not recorded in both programs. We conservatively assumed all patients underwent laboratory monitoring as per the South African public-sector guidelines. The guidelines recommended six monthly CD4 and VL monitoring. Laboratory toxicity monitoring, which occurred predominant in the first six months on ART, was limited to ZDV, NVP, and LPV/r. We scaled the specific toxicity monitoring utilisation associated with a specific ARV drug in accordance with its relative proportion within the two regimen lines. All laboratory costs were set at the public sector tender prices for April 2010. All costs were converted from ZAR to United States Dollars (USD) in April 2010 (7.34 ZAR per USD).

### The Markov model framework, development and uncertainty analysis

WHO stage, current CD4, and current VL were identified as key determinants of lifetime costs and outcomes [9]. Many patients categorized as “LTFU” in studies return to ART care and therefore are not truly LTFU [10]. This is important as: (a) ART-related resources are not consumed while a patient is LTFU, (b) the CD4 count falls rapidly to pre-ART levels in patients who interrupt ART [11], (c) additional resources are consumed in patients restarting ART [9], (d) treatment interruptions increase resistance to first line regimens [9] [9] [9] [9] [9](9)(9)(8), and (e) treatment interruptions increase deaths [9] and attenuate CD4 recovery [12].

We based the structure of the Markov model on these determinants of costs and outcomes as well as on our own analysis of the public-care program – the larger of the two cohorts. We implemented this Markov model in Treeage 2009 [13] and populated it with parameter estimates derived in Stata 11 [14] using survival models for time-to-event analyses and generalized linear models for clinic/GP utilisation. We evaluated the model fit and adjusted the model design where appropriate. Then, using the data from the private-care program, we derived new parameter estimates and evaluated the ability of the model to predict outcomes and costs. This procedure allowed us to assess the external validity of the model [4], [15]. The model was run for two durations: 10 years and until all members of each cohort were dead (i.e. lifetime duration). Finally, we conducted probabilistic sensitivity analysis to assess uncertainty. This entailed specifying distributions on utilization and outcome parameters, where possible and propagating uncertainty through the model by way of first and second order Monte Carlo simulations. The models were run using a 1 month cycle length [16], [17].

### The Markov Model

**Figure 1:**
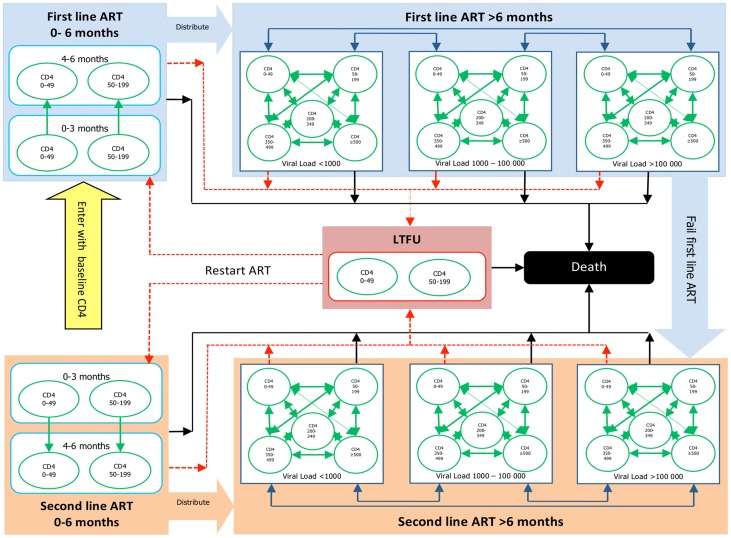
Markov model diagram.

The overall Markov model was divided into two parts: an ART model and a LTFU model (see figure 1). All patients started in the ART model, and remained there until they either died or became LTFU. Healthcare utilisation and mortality has been shown to be significantly higher in the first 6 months on ART [4], [6]. Therefore the ART model was divided into two phases: 0–6 months on starting or restarting ART and >6 months on ART. We defined LTFU as defaulting ART for more than 6 months. Patients entering the LTFU model remained there until they either died or restarted ART. We used parametric survival analysis with an exponential distribution to determine the transition probabilities to outcomes (death, LTFU, CD4 category change, and VL category change), and generalized regression models to determine utilisation (GP and clinic visits) within the Markov states. Covariates included time on ART, on-ART CD4 category, on-ART VL category, and year of starting ART (normalizing findings to 2005). We assumed that non-HIV related deaths of a typical individual (34 years) were included in the recorded deaths. We modeled the increasing relative contribution of non-HIV related deaths over time using the mortality curves for South Africa (less the typical mortality for a 34 year old adult) before the onset of South Africa's HIV epidemic (prior to 1990).

In the first 6 months after starting or restarting ART, patients were split according to their pre-ART CD4 count category (0–49 or 50–199 cells/µL), and remained within this CD4 category for 6 months. At the end of 6 months, the remaining patients (i.e. not LTFU or dead) were distributed into the Markov states of the >6 months on ART model using a competing risks regression model with the pre-ART CD4 category as the only covariate. The >6 months on ART phase was defined by fifteen Markov states. These included: five on-ART CD4 categories (0–49, 5–199, 200–349, 350–499, and ≥500 cells/µL) and three on-ART VL categories (<1,000; 1,000–99,999; and ≥100,000 copies/mL). Within each Markov cycle, we limited transitions between these Markov states to either a CD4 or VL category change but not both, as this reduced model complexity.

We distributed patients entering the LTFU model into the two pre-ART CD4 categories (0–49 and 50–199 cells/µL) with the relative proportions being derived from the observed data. Given the limited LTFU data within our cohorts, we used the transition probability from the higher to the lower pre-ART CD4 category on a previously published natural history HIV model [4], and adapted the transition probabilities from these CD4 categories to death to match the observed trends in deaths within our cohorts. We used a regression model to determine the transition probability of restarting ART for patients LTFU, with time since first starting ART as the covariate.

The transition probability from first line to second line ART was determined separately within the two phases of the ART model and the covariates for the regression model included pre-ART CD4 category, on-ART VL category, on-ART CD4 category, and time since starting ART. Within the second line ART model all transition probabilities were the same as the first line ART model, but the ARV drug utilisation and therefore costs differed. Patients within the LTFU model were assigned no ART-related utilisation and therefore no costs.

### Uncertainty analysis

We assessed the uncertainty in the data and model design using probabilistic sensitivity analysis (first and second-order Monte Carlo simulations). First-order simulations were used to capture the variability in the simulated population and tracked the varying paths taken by patients moving through the model in order. Second-order simulations were used to capture the variability in the parameter estimates by randomly sampling from the triangular-shaped distribution for the parameter, which approximated the 95% confidence interval. We ran 1,000 second-order and 10,000 first-order simulations to determine the 95% uncertainty intervals around the lifetime costs and outcomes. We assessed uncertainty related to extrapolation of the data and the generalizability of the model in three ways: (1) we externally validated the model derived from public-care cohort using the private-care cohort dataset, (2) we extrapolated our estimates over 10 year and life-time durations and compared the results, and (3) we compared our outcomes and cost estimates with other published studies. Finally, we assessed the uncertainty related to analytical methods by comparing the findings with 0% and 3% annual discounting of costs and outcomes.

### Scenario analysis

Clinic visit utilisation within the public-care program was intensive due to a policy decision by the program managers that all patients should be seen by a nurse or doctor every one to two months. In more recent years, the clinic visit utilisation has been substantially reduced to accommodate the growing number of patients. We therefore explored the impact of reduced clinic visit utilisation within the public-care program on the overall results.

### Ethics statement

The study was approved by the Research Ethics Committee, University of Cape Town. All patients signed consent for their information to be entered into the central databases and analysed. Anonymity was ensured using generated identifiers and all personal data were deleted from the datasets.

## Results

### Cohorts

The characteristics and overall outcomes of the study cohorts are described in Table 1. We included 6372 and 963 patients from the public-care and private-care programs respectively. Median follow-up time on ART was shorter in the public-care cohort. No patients were transferred out to other facilities from the private-care program. The model fit diagnostics for both the private-care and public-care programs are shown in figures S1 and S2 respectively. These include current CD4, current VL, line of therapy and status (current, LTFU or dead).

**Table 1:** Cohort characteristics.

Characteristic	Khayelitsha	Broadreach
Numbers	6372	963
Age baseline (years)		
Median	33	34,9
IQR	(28,7 to 39,3)	(30,4 to 41,9)
Sex (%)		
Female	67,7	68,3
CD4 count (cells/µl) baseline		
Median	99	92
IQR	(44 to 161)	(44 to 146)
Unknown	435	3
Viral load (log_10_) baseline		
Median	5,1	5,1
IQR	(4,6 to 5,6)	(4,7 to 5,6)
Unknown	2941	241
Follow-up duration (months)		
Median	21,3	54,6
IQR	(11,7 to 33,4)	(29 to 57,8)
Status at end of study (%)		
Current	77,5	72,2
Transferred	6,3	0
LTFU	5,5	7,8
Deceased	10,6	20

### Health care utilization and unit costs in Markov states

Over the study period, 212,175 clinic visits in the public-care cohort and 10,477 GP visits in the private-care cohort were recorded. The contracted rate for a GP visit was 31.04 USD and the estimated cost of 24.53 USD for a clinic visit was derived by inflating the cost estimate from a previous publication [4]. The average monthly GP/clinic utilisation (with 95% confidence intervals) and the cost estimates are shown in table S1. Within both cohorts, utilisation was highest in patients restarting ART and, to a lesser extent, during the 0–6 months after starting ART, compared with the >6 months on ART phase. In this latter phase, monthly visit utilisation was lower in both cohorts. Importantly, the public-care cohort had approximately 2 to 4 times higher visit utilisation within the >6 months on ART phase compared with the private-care cohort.

The South African public sector guidelines were used for laboratory utilisation – the costs and utilisation are shown in table S2. CD4 and VL were taken 6 monthly, whilst other laboratory utilisation related to toxicity monitoring depended on the specific antiretroviral drugs and was higher in the first 6 months on ART.

The utilisation of individual drugs within the first and second line ART regimens, the ART-related costs, and the hazard coefficients and transition probabilities for the model describing the transition between first and second line ART are shown in table S3 and figure S3. We assumed 100% utilisation of both ARV drugs and laboratory tests while within the ART model. The public-care cohort had higher zidovudine but lower efavirenz utilisation in the first line ART regimen. The public-care cohort had higher didanosine utilisation in the second line ART regimen. The transition probability of moving to second line ART was lowest in the 0–6 months after starting ART and highest in the 0–6 months after restarting ART. In the >6 month on ART phase, the transition probability of moving to second line ART decreased with lower VL and higher CD4 categories respectively, increased with time on ART and plateaued at about 3 years. The transition probabilities to second line ART were generally lower in the private-care cohort. The estimated distribution of time between first and second line ART was 61% and 39% in the public-care cohort versus 66% and 34% in the private-care cohort.

### Effectiveness

The transition probabilities for the CD4 and VL models on ART are shown in table S4. The baseline CD4 category distribution for patients starting ART was similar in both cohorts: 30% in the 0–49 cells/µL category and 70% in the 50–199 cells/µL category. A lower baseline CD4 category was associated with a lower CD4 category distribution after 6 months on ART, but lower baseline CD4 category did not impact on the VL distribution. Public-care patients were more likely than private-care patients to have VL <1000 copies/ml (92% versus 87%) and CD4 counts ≥200 cells/µL (64% versus 42%) after the first 6 months on ART. This trend was similar for patients restarting ART, but the outcomes were worse: 61% and 43% had VL<1000 copies/ml, and 49% and 63% had CD4 counts <200 cells/µL for patients in the public-care and private-care cohorts respectively.

The transition probabilities and hazard coefficients for deaths on ART are shown in table 2. The transition probability to death was highest in the first 3 months on ART and in patients with a low pre-ART CD4 category. The transition probability to death was lowest for the first 6 months after restarting ART. For patients in the >6 months on ART phase, the transition probability to death decreased with lower VL category, higher CD4 category, and time on ART (using a Gompertz time function). The median of the Gompertz time function was 20 months in both cohorts, but the scaling constant was higher in the private-care cohort (1.19 versus 1.04). Thus there were more early deaths in the private-care cohort.

**Table 2:** Transition probabilities and hazard coefficients for deaths on antiretroviral therapy.

Variables		Transition probabilities and hazard coefficients (95% CI) per 1 month cycle	
		Public-care	Private-care
**First 6 months after starting antiretroviral therapy**			
**Transition probability**			
3 months CD4 0–49 cells/µL		0,035 (0,029 to 0,044)	0,040 (0,029 to 0,056)
3 months CD4 50–199 cells/µL		0,010 (0,008 to 0,012)	0,017 (0,013 to 0,022)
6 months CD4 0–49 cells/µL		0,011 (0,010 to 0,014)	0,027 (0,021 to 0,036)
6 months CD4 50–199 cells/µL		0,003 (0,003 to 0,004)	0,011 (0,009 to 0,014)
**First 6 months after restarting antiretroviral therapy**			
Transition probability: 0–6 months		0,008 (0,004 to 0,016)	0,004 (0,001 to 0,010)
**>6 months on antiretroviral therapy**			
**Hazard coefficient due to CD4 and VL**			
CD4 0–49 cells/µL	VL <1,000 copies/ml	−5,01	−5,03
CD4 0–49 cells/µL	VL 1,000–100,000 copies/ml	−4,71	−4,69
CD4 0–49 cells/µL	VL >100,000 copies/ml	−3,83	−4,13
CD4 50–199 cells/µL	VL <1,000 copies/ml	−6,00	−6,5
CD4 50–199 cells/µL	VL 1,000–100,000 copies/ml	−5,69	−6,16
CD4 50–199 cells/µL	VL <1000 copies/ml	−4,82	−5,6
CD4 200–349 cells/µL	VL >100,000 copies/ml	−7,25	−7,48
CD4 200–349 cells/µL	VL 1,000–100,000 copies/ml	−6,94	−7,14
CD4 200–349 cells/µL	VL <1000 copies/ml	−6,07	−6,58
CD4 350–499 cells/µL	VL >100,000 copies/ml	−7,63	−8,53
CD4 350–499 cells/µL	VL 1,000–100,000 copies/ml	−7,32	−8,19
CD4 350–499 cells/µL	VL >100,000 copies/ml	−6,45	−7,63
CD4 ≥500 cells/µL	VL <1,000 copies/ml	−7,76	−8,16
CD4 ≥500 cells/µL	VL 1,000–100,000 copies/ml	−7,46	−7,82
CD4 ≥500 cells/µL	VL >100,000 copies/ml	−6,58	−7,26
**Hazard coefficients for Gompertz function**			
alpha		0,93 (0,52 to 1,34)	1,73 (1,17 to 2,28)
beta – half-life (months)		20	20

The hazard coefficients and transition probabilities related to the LTFU model are shown in table 3. The transition probability from ART to LTFU was lowest in the first 6 months after starting ART and highest in the first 6 months after restarting ART. Thereafter, the transition probability from ART to LTFU increased with higher VL category, lower CD4 category, and time on ART. We modeled the effect of time on ART by adapting the Gompertz function so that it plateaued. The median of the adapted Gompertz function was longer (12 months versus 8) and the scaling constant has higher (1.5 versus 0.5) in the public-care compared with the private-care cohort. We distributed patients entering the LTFU model as follows based on our analysis of the data: 30% to the 0–49 cells/µL and 70% to the 50–199 cells/µL CD4 categories. The transition probability from LTFU to restarting ART was higher in the private-care cohort (26% versus 13%) and independent of LTFU CD4 category.

**Table 3:** Transition probabilities and hazard coefficients related to loss to follow-up.

Variables	Transition probabilities and hazard coefficients (95%) per 1 month cycle			
	Public-care			Private-care
**Transitions within ART model**				
**Transition probability to LTFU within 0–6 months on ART**				
On starting ART		0,0085 (0,0080 to 0,0091)		0,0006 (0,0006 to 0,0006)
On restarting ART		0,0270 (0,0205 to 0,0356)		0,0251 (0,0251 to 0,0251)
**Hazard coefficient to LTFU within >6 months on ART**				
CD4 0–49 cells/µL	VL <1,000 copies/ml	−4,7		−5,13
CD4 0–49 cells/µL	VL 1,000–100,000 copies/ml	−3,79		−4,16
CD4 0–49 cells/µL	VL >100,000 copies/ml	−4,00		−4,37
CD4 50–199 cells/µL	VL <1,000 copies/ml	−5,31		−5,44
CD4 50–199 cells/µL	VL 1,000–100,000 copies/ml	−4,4		−4,47
CD4 50–199 cells/µL	VL <1000 copies/ml	−4,61		−4,68
CD4 200–349 cells/µL	VL >100,000 copies/ml	−5,73		−4,52
CD4 200–349 cells/µL	VL 1,000–100,000 copies/ml	−4,82		−3,56
CD4 200–349 cells/µL	VL <1000 copies/ml	−5,03		−3,76
CD4 350–499 cells/µL	VL >100,000 copies/ml	−5,73		−4,52
CD4 350–499 cells/µL	VL 1,000–100,000 copies/ml	−4,82		−3,56
CD4 350–499 cells/µL	VL >100,000 copies/ml	−5,03		−3,76
CD4 ≥500 cells/µL	VL <1,000 copies/ml	−5,73		−4,52
CD4 ≥500 cells/µL	VL 1,000–100,000 copies/ml	−4,82		−3,56
CD4 ≥500 cells/µL	VL >100,000 copies/ml	−5,03		−3,76
**Hazard coefficients for Gompertz function**				
alpha			1,5	0,5
beta – half-life (months)			12	8
**Initial distribution within LTFU model**				
CD4 0–49 cells/µL			0,278 (0,255 to 0,302)	0,243 (0,217 to 0,269)
CD4 50–199 cells/µL			0,722 (0,745 to 0,698)	0,757 (0,783 to 0,731)
**Transitions within LTFU model**				
**Transition probability between CD4 category**				
CD4 50–199 to CD4 0–49 cells/µL			0,005 (0,005 to 0,005)	0,006 (0,006 to 0,006)
**Transition probability back to ART**				
CD4 0–199 cells/µL			0,134 (0,128 to 0,141)	0,146 (0,139 to 0,154)
**Transition probability to death**				
CD4 0–49 cells/µL			0,006 (0,005 to 0,008)	0,006 (0,005 to 0,008)
CD4 50–199 cells/µL			0,001 (0,001 to 0,017)	0,001 (0,001 to 0,017)

The highest death rates were observed within the first year on ART for both cohorts, especially in the private-care cohort: 8% and 15% had died by 12 months and 32% and 39% had died by 120 months in the public-care and private-care cohorts respectively. The distribution of VL categories stabilized by 3 years to 90% and 85% of patients having a VL <1000 copies/ml within public and private-care cohorts respectively. The distribution of CD4 categories was more dynamic over time and the private -care cohort fared better with 50% versus 40% of patients having a CD4 ≥500 cells/µL by 10 years. The percentage of patients who were alive and still on ART stabilized at approximately 80% for both cohorts, although the private-care cohort achieved this earlier due to generally higher transition probabilities to and from LTFU.

### Ten**-**year and lifetime costs, outcomes, probabilistic sensitivity and scenario analysis

We ran Monte Carlo simulations for 10 years and until everyone had died to generate lifetime costs and outcomes together with their 95% confidence intervals, as shown in table 4. The conclusions we derived from the 10 year and lifetime estimates (with and without discounting) were congruent: the private-care program was approximately as effective, but was less costly than the public-care program. These reduced costs were predominantly driven by the lower level of utilisation in the private-care program. Given that the outcomes between the two programs were not significantly different, this finding suggests that reduced visit utilization has the potential to be cost saving (reducing costs without impacting on patient outcomes).

**Table 4:** 10 year and lifetime estimates of cost and outcomes of the private-care and public-care programs.

Treatment option	10 year estimates		Lifetime estimates	
	Costs (95% CI) in USD	Life years gained (95% CI)	Costs (95% CI) in USD	Life years gained (95% CI)
**Undiscounted**				
Public-care	8,825 (8,614 to 9,036)	7.6 (7.4 to 7.8)	18,734 (17,385 to 20,083)	14.1 (13.2 to 15.0)
Private-care	6,187 (5,997 to 6,377)	7.2 (7.0 to 7.4)	13,062 (12,077 to 14,047)	14.0 (13.1 to 14.8)
**Discounted**				
Public-care	7,688 (7,513 to 7,863)	6.7 (6.5 to 6.8)	13,305 (12,588 to 14,022)	10.4 (9.9 to 10.9)
Private-care	5,407 (5,250 to 5,564)	6.3 (6.2 to 6.5)	9,273 (8,704 to 9,842)	10.0 (9.4 to 10.5)

When we reduced the frequency of clinic visits in the >6 months on ART phase by two-thirds in the public-care program (in line with the changes introduced in late 2011 by the program administrators), the estimated 10-year and lifetime costs within the public-care program approximated the levels observed in the private-care program. In other words, the programs were equivalent in terms of costs and outcomes.

## Discussion

We determined that the private-care program had lower costs and similar outcomes to the public-care program at the time of the study using a novel Markov model. Key differences between the programs were less frequent visits and higher rates of returning to care after loss to follow-up in the private-care program, and lower early death rates on ART, but more deaths while LTFU in the public-care program. We estimated that the recent shifts towards less frequent visits in the public-care ART program would achieve large cost savings, making the costs of the two programs similar. These findings suggest that properly managed private-care programs can ease the burden of ART care in endemic countries by looking after public sector patients without increasing costs. Further, reducing clinic visits may be a viable strategy to save costs while maintaining outcomes in public sector programs.

Our Markov model included several significant improvements on previously published models [4], [18]–[22]. First, we separated out the first six months on ART, as outcomes and costs in this period are driven by baseline CD4 count and program protocols (higher frequency of clinic visits and toxicity monitoring) [6]. Second, we developed a novel LTFU model, in which patients transitioned between ART and LTFU, changed baseline CD4 count within LTFU, and transitioned to death within LTFU. Third, we developed Markov models to account for CD4 and VL category changes within the ART and LTFU models. Fourth, we developed a more detailed model describing the transition between first line and second line ART, which is a major cost driver [23]. Fifth, the model included the impact of time on ART on the transition to LTFU, death, and second line ART. Finally, we assessed the external validity of the model by first developing the model using the public-care program data and then validating it using private-care program data. The fact that our novel Markov model was able to describe the data from two very different models of ART care suggests that its utility may be generalizable.

We are aware of one other study that compared costs and outcomes after 1 year in public-care and private-care programs for public sector patients [24]. Their private-care program had significantly lower costs due to fewer GP visits and poorer patient retention than their public care program. The costs of providing ART care were similar, although patient retention was better in our programs. Lifetime analyses using Markov models populated with data from resource-limited settings predicted varying survival on ART (6 to 13 years) and varying discounted total costs (3,000 to 9,500 USD from the provider's perspective) [16], [18], [21]–[23], [25], [26]. Many of these models were developed using short term follow up data. Furthermore, retention within ART programs and cost of providing ART care in resource-limited settings varies dramatically [27], [28]. We estimated that average survival on ART was longer than most resource-limited setting model estimates.

The patients included in this analysis were public-sector patients receiving ART care in accordance with WHO public sector ART program guidelines. Therefore the results from this analysis have important policy implications that are relevant to other resource-limited settings. The rapid expansion of access to ART in resource-limited settings is both needed [29] and challenging [30]. Our findings suggest that managed private-care for public sector patients could be used to increase access to ART, provided that the private practices follow national protocols and that loss to follow-up is managed – key components of the private-care program in our study. A similar model was implemented in Botswana to expand access to ART in areas where limited public-sector resources were available, by utilising doctors working in private practice to look after public sector patients [2]. Their findings suggested that ART care coverage was extended by 10% and public-sector programs were strengthened by the interaction [2]. We found that reduced utilisation of clinic visits, especially after the initial six months of care, would considerable lower costs of public-care programs. Finally, our model predicted that LTFU contributed significantly to deaths, utilisation of ART-related resources (on restarting ART), and attenuated CD4 recovery. This suggests that focusing on reducing LTFU could be a cost-saving strategy.

There were several limitations to our study. First, the findings in our study are based on a model that extrapolated the trends we observed over the first 3–5 years on ART predominantly. Second, we limited costs in this study to direct ART care costs, while the other components of care represent a significant portion of total costs [31]. Data on these other cost components were not available. Third, we did not account for the impact of adherence on the total cost of ART drugs, nor the changing composition of specific drugs within the therapy lines over time [31], [32]. Fourth, given the limited data on actual laboratory utilisation, especially for toxicity monitoring, we set the laboratory utilisation to those recommended in national guidelines. Fifth, it is likely that the patients within the public-care program had better access to HIV clinic services than typical public-sector patients in South Africa, and this would have increased costs, and possibly enhanced patient retention and improved outcomes [33]. Sixth, the relative proportions of individual drugs within the lines of therapy differed between cohorts: *the average ART costs were marginally lower in the private-care program and the different regimens may have impacted the outcomes*. Seventh, given the different models of ART care and different settings in which the programs were based, these programs were not completely comparable and therefore the overall conclusions in terms of costs and outcomes cannot be regarded as definitive. Finally, our public sector clinic visit cost was based on secondary data, which may not capture recent programmatic changes in ART provision (including task shifting) and economies of scale and scope. However, it is difficult to predict the extent to which this unit cost may under or overestimate costs. In moving towards universal access to ART, South Africa intends to offer ARVs from all primary care facilities, which will have implications for the efficiency of service provision and the resulting unit cost. Economies or diseconomies of scale can equally arise in small new facilities during start-up and in older large facilities with high patient volumes.

While analyses of provider costs and patient outcomes are crucial in guiding resource allocation for HIV care, it is equally important to consider barriers to patient access, particularly within the context of lifelong care [34]. Evidence suggests that the key barriers to ongoing ART care include the cost of transport to facilities as well as the opportunity cost associated with long waiting times in facilities [34], [35]. Less frequent visits would mitigate these access barriers. One advantage of private care is that waiting times are usually shorter.

### Conclusions

In conclusion, we have developed a novel Markov model that has the potential to improve the accuracy of estimations of future costs and outcomes of long-term ART care. We have used this model to evaluate two ART programs, and have shown that managed private-care ART programs have the potential to complement the public sector platform in resource poor settings, thereby enhancing and sustaining coverage of patients in need. Our findings suggest that cost savings could be achieved through reducing clinic utilization without compromising patient outcomes.

## Supporting Information

Figure S1 **Model calibration curves for the public-care ART program.**
(TIF)

Figure S2 **Model calibration curves for the private-care ART program.**
(TIF)

Figure S3 **Survival hazard coefficient for switching from first to second line therapy over time since starting antiretroviral therapy.**
(TIF)

Table S1 **General practitioner and clinic visit utilisation and costs on antiretroviral therapy within the private-care and public-care programs respectively.**
(XLS)

Table S2 **Laboratory costs and utilisation on antiretroviral therapy.**
(XLS)

Table S3 **The composition and costs of first line and second line antiretroviral regimens, and transition probabilities and coefficients for transitioning from first line to second line regimen.**
(TIF)

Table S4 **Transition probabilities and hazard coefficients of changes in CD4+ cell counts and viral load on antiretroviral therapy.**
(XLS)
