# Supplementary figures and images for: A Novel Markov Model Projecting Costs and Outcomes of Providing Antiretroviral Therapy to Public Patients in Private Practices versus Public Clinics in South Africa
Source: PLoS One. 2013 Feb 6;8(2):e53570. doi: 10.1371/journal.pone.0053570 (PMC3566152; doi:10.1371/journal.pone.0053570)

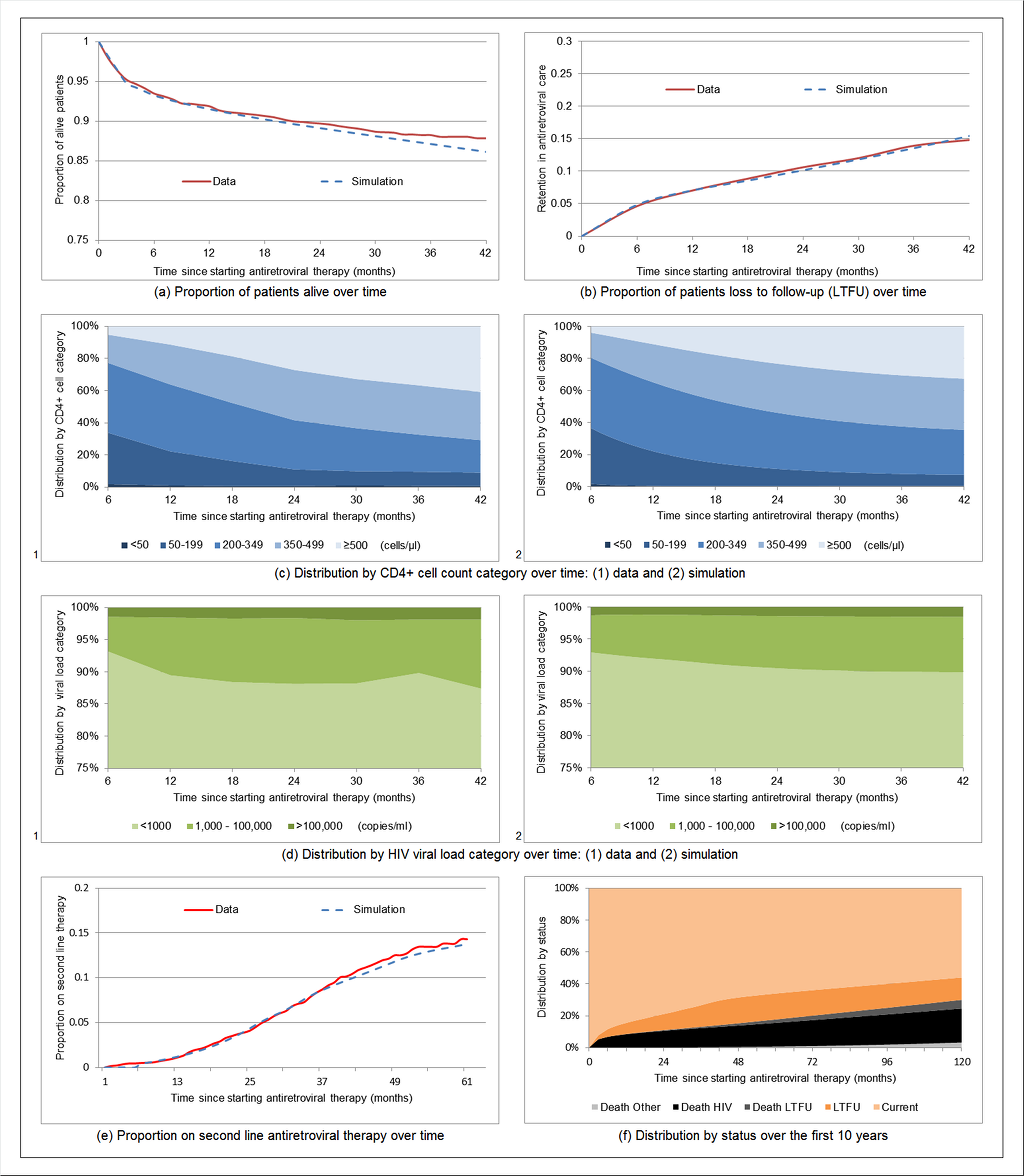

Supplement: Figure S1 — Model calibration curves for the public-care ART program. (TIF) [file pone.0053570.s001.tif]

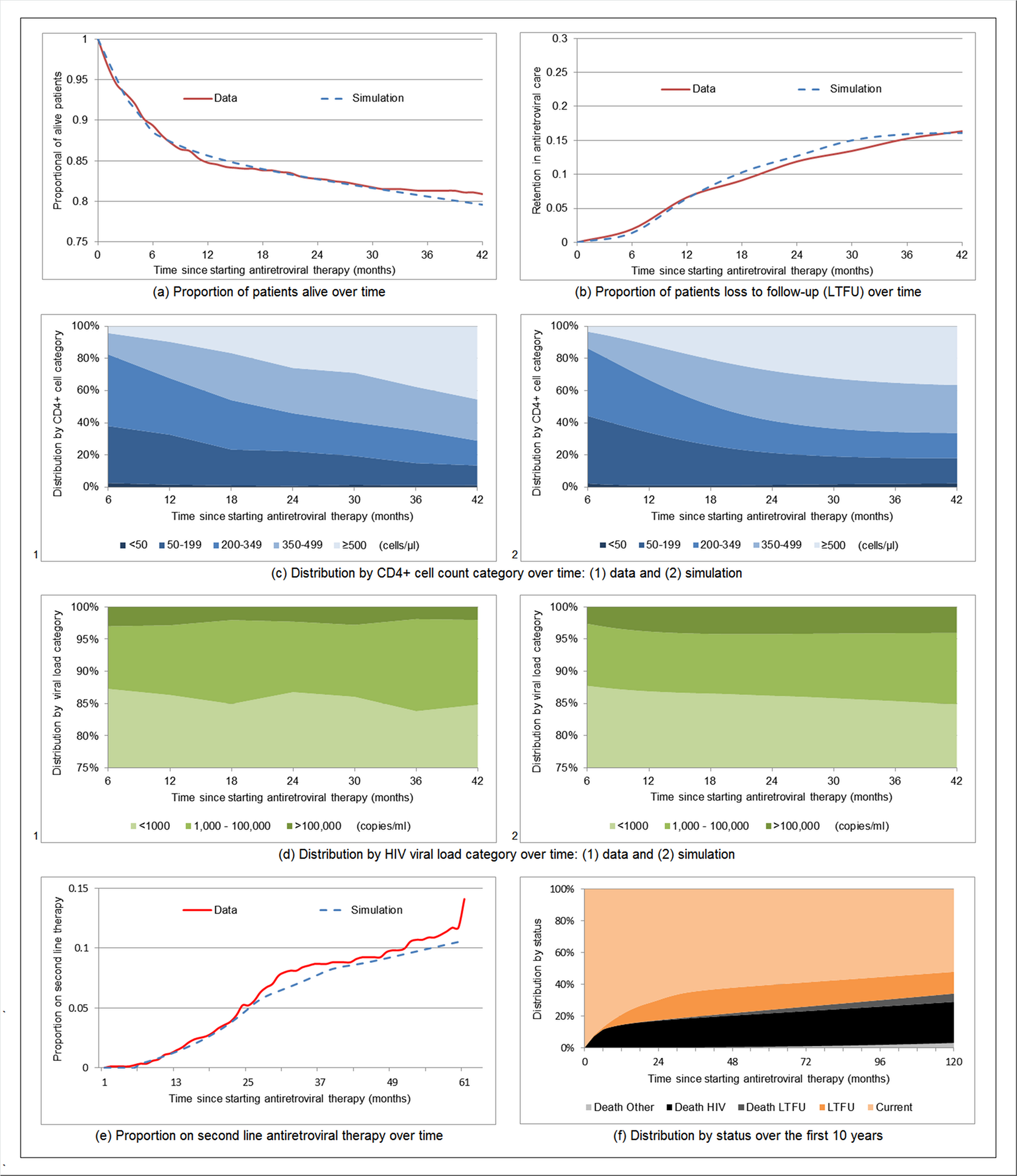

Supplement: Figure S2 — Model calibration curves for the private-care ART program. (TIF) [file pone.0053570.s002.tif]

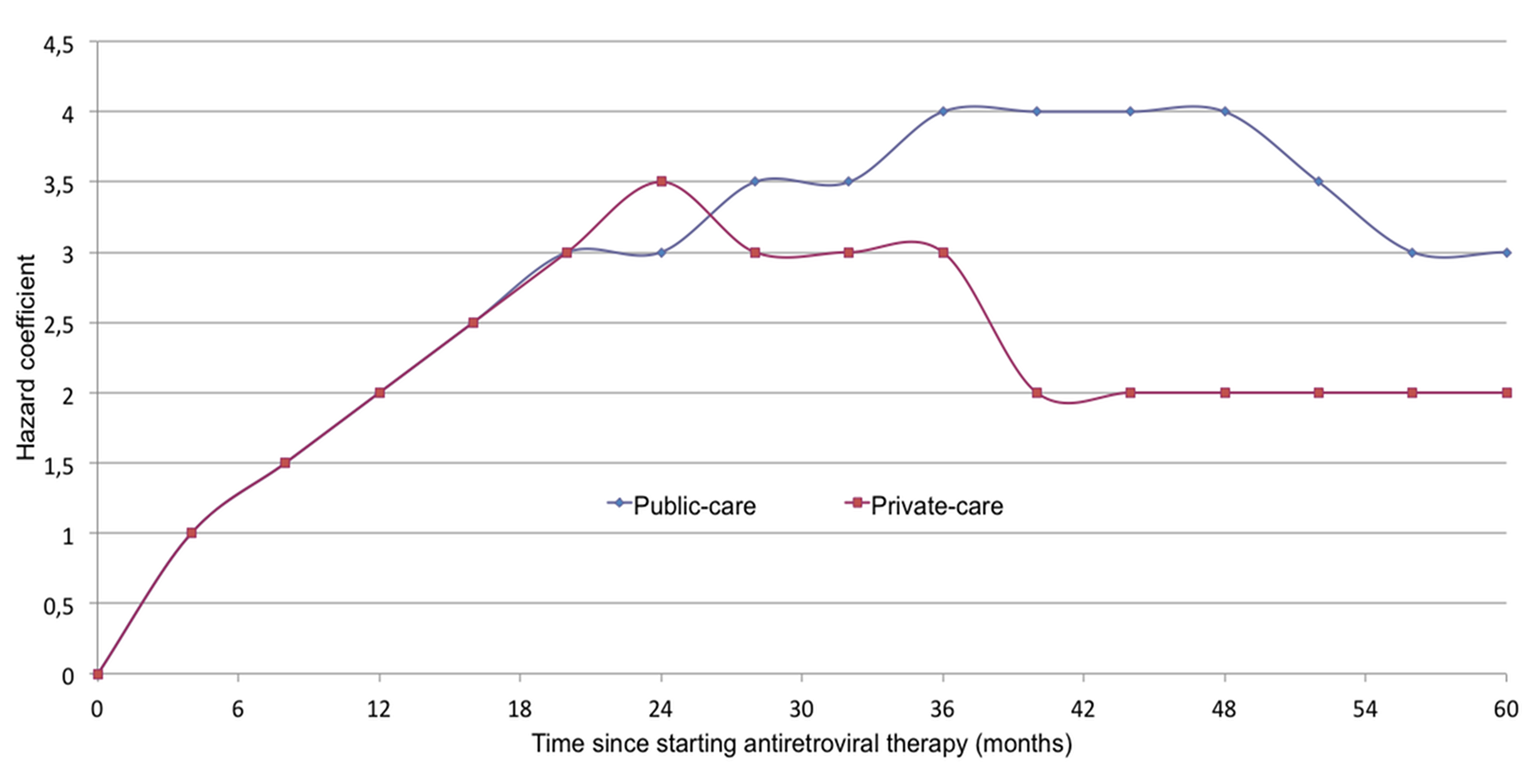

Supplement: Figure S3 — Survival hazard coefficient for switching from first to second line therapy over time since starting antiretroviral therapy. (TIF) [file pone.0053570.s003.tif]

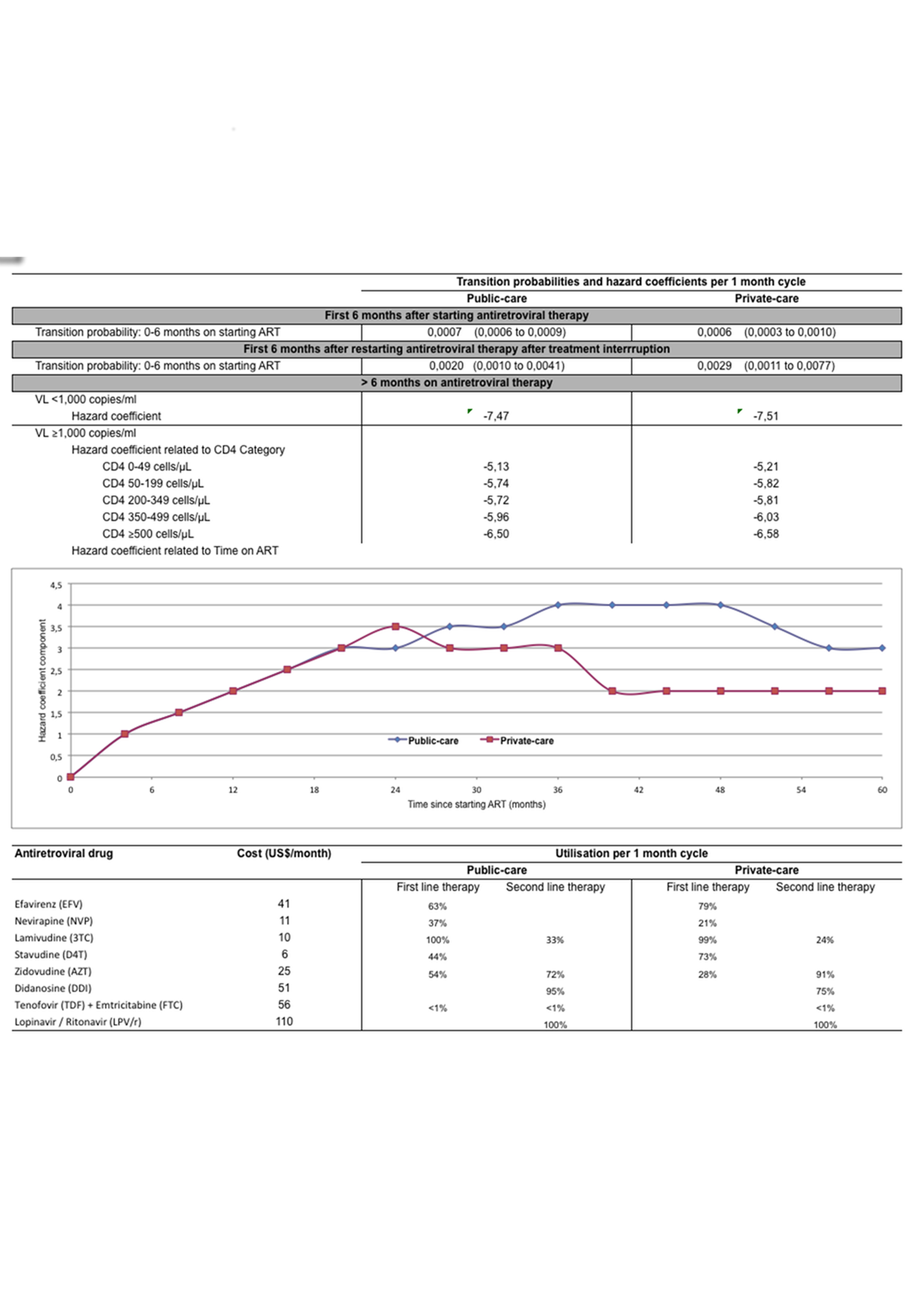

Supplement: Table S3 — The composition and costs of first line and second line antiretroviral regimens, and transition probabilities and coefficients for transitioning from first line to second line regimen. (TIF) [file pone.0053570.s006.tif]
